# George Gilbert Currey

**Published:** 1823-01

**Authors:** 


Died, lately, George Gilbert Currey, m.d. senior physician to St. Thomas's Hospital. We understand that Dr. Currey had for some time laboured under an affection of the heart, and that the symptoms of his last illness were referrible to the chest. He had been very lately married.

